# NEDD 4 Pyre-fighters to extinguish inflammation: NEDD4L ubiquitinates Gasdermin D and Gasdermin E to dampen pyroptosis

**DOI:** 10.1038/s41418-025-01626-0

**Published:** 2025-12-09

**Authors:** Kailash Gulshan

**Affiliations:** 1 Center for Gene Regulation in Health and Disease, Cleveland State University, Cleveland, OH USA; 2 Department of Biological, Geological, and Environmental Sciences, Cleveland State University, Cleveland, OH USA; 3 Department of Cardiovascular and Metabolic Sciences, Lerner Research Institute, Cleveland Clinic, Cleveland, OH USA

**Keywords:** Macroautophagy, Immunological disorders

Since the identification of Gasdermin D (GSDMD) as a pyroptosis-inducing substrate of inflammatory caspases [1, 2], the field of cell death research has been captivated by the dramatic spectacle of pyroptosis, with studies showing that this cell-shredding process, characterized by cell swelling and membrane rupture, is ingrained in the body’s response to various physiological and pathological cues. Gasdermin (GSDM) family, consisting of six proteins—GSDMA, GSDMB, GSDMC, GSDMD, GSDME (DFNA5), and DFNB59 (PJVK). All members, except DFNB59, can undergo proteolytic cleavage by various caspases. GSDMD, the most studied family member, is cleaved by inflammatory caspases (caspase-1, -4, -5, and mouse caspase-11) and neutrophil proteases like elastase and cathepsin G. It consists of two domains, the 31-kDa N terminus (GSDMD-N) and the 22-kDa C terminus (GSDMD-C), separated by a linker region [3]. The cleaved N-terminal fragment oligomerizes at the plasma membrane via binding to phosphatidylinositol phosphates (PIPs) or phosphatidylserine (PS), or at the mitochondrial membrane via binding to cardiolipin [4], leading to membrane pores. These pores allow the release of highly inflammatory cytokines and other inflammatory molecules. GSDME can be activated by apoptotic caspase-3 and granzyme B from cytotoxic lymphocytes, often converting apoptosis into a more inflammatory pyroptotic cell death.

The notion of Gasdermin proteins as dormant executioners awaiting a single proteolytic cue has been dismantled by the inundation of recent research. We now understand that a complex web of regulatory mechanisms governs the fate and function of these proteins, with succination [5] and palmitoylation [6] emerging as central orchestrators. Other modifications, such as ubiquitination, phosphorylation, oxidation, acetylation, and SUMOylation, also modulate the pyroptotic activity of Gasdermin proteins. The latest findings on the ubiquitination of GSDMD and GSDME highlight the critical, and somewhat peculiar, roles played by HECT and RING E3 ligases in modulating their stability and pyroptotic activity. Recent studies have identified specific E3 ligases that positively and negatively regulate GSDMD through ubiquitination, demonstrating the complexity of this regulatory cascade. One of the intriguing discoveries is the identification of SYVN1, a RING E3 ligase, as a positive regulator of GSDMD-mediated pyroptosis [7]. Mechanistically, SYVN1 directly interacts with GSDMD and catalyzes its polyubiquitination. This modification, contrary to the typical expectation of proteasomal degradation, serves to promote GSDMD’s pyroptotic activity. This finding challenges the conventional wisdom of ubiquitination as a purely degradative signal and showcases the diverse functions of different polyubiquitin linkages. The promotion, rather than dampening, of pyroptosis by SYVN1/GSDMD ubiquitination may be an integral part of cellular adaptations to amplify the inflammatory signal, ensuring a robust and timely response to pathogens.

**Fig. 1:**
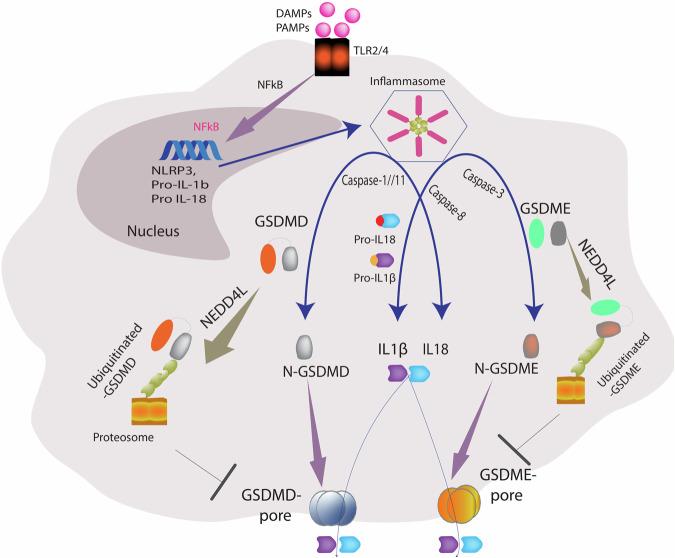
NEDD4L regulates pyroptosis by ubiquitinating GSDMD and GSDME. Schematic diagram showing TLR2/4 activation by danger-associated molecular patterns (DAMPs) or Pathogen-associated molecular patterns (PAMPs), nuclear localization of NF-kb, and transcription of NLRP3 and inflammatory cytokines. Inflammasome assembly leads to GSDMD and GSDME cleavage by different caspases, leading to the release of pore-forming N-terminal fragment and the release of mature inflammatory cytokines. Ubiquitination of GSDMD/GSDME by NEDD4L leads to their proteasomal degradation, preventing membrane pores and pyroptosis. Image created using Adobe Illustrator.

Interestingly, components of inflammasome assembly, such as NLRP3, are known to be negatively regulated by ubiquitination [8], but are there E3 ligases involved in regulating GSDMD and GSDME? A recent study (Shah et al., NEDD4L mediated Gasdermin D and E ubiquitination regulates cell death and tissue injury, Cell Death Differ. 2025: in press) in the journal “Cell Death and Differentiation” linked the E3 ligase activity of NEDD4L to ubiquitination-mediated degradation of GSDMD and GSDME (Fig. 1). Interestingly, Nedd4l KO mice demonstrated elevated GSDMD in alveolar epithelia and increased GSDME in kidney tubular epithelia, suggesting tissue-specific regulation of Gasdermin proteins by NEDD4L (Shah et al., NEDD4L mediated Gasdermin D and E ubiquitination regulates cell death and tissue injury, Cell Death Differ. 2025: in press). NEDD4L-deficient cells were significantly more susceptible to GSDM activation and showed increased cell death by NLRP3 agonists, cytotoxic agents, and bacterial infection, and higher IL-1β release. NEDD4L binds and ubiquitinates both GSDMD and GSDME, targeting them for proteasomal degradation (Shah et al., NEDD4L mediated Gasdermin D and E ubiquitination regulates cell death and tissue injury, Cell Death Differ. 2025: in press). GSDMD N-terminal fragment gets ubiquitinated at K51, K203, and K204, while GSDME was ubiquitinated at K39, K40, and K120 in the N-terminal domain and at K440 in the C-terminal domain. This reveals a feedback loop mechanism to prevent excessive and damaging inflammatory responses. Variants in human NEDD4L are associated with developmental disorders, hypertension, and end-stage renal disorders, while NEDD4L KO mice show disorders of the respiratory, renal, cardiac, neural, and immune systems [9], and aberrant GSDMD/GSDME activity may be one of the mechanisms promoting these disease manifestations.

These findings underscore the subtle and multifaceted control over GSDMD, where different ligases and ubiquitin linkages can either promote or inhibit its function, offering layers of regulation to maintain cellular homeostasis.

The functional differences between HECT and RING E3 ligases may provide a fascinating framework for understanding the contradictory regulatory mechanisms behind Gasdermin ubiquitination. HECT ligases, known for their two-step reaction mechanism involving a catalytic cysteine intermediate, can specify a diverse range of polyubiquitin chains, including non-canonical linkages like K27. A HECT ligase, such as NEDD4L, could target GSDMD or GSDME for degradation via K48-linked and other polyubiquitinations, providing a separate rheostat for controlling protein levels distinct from the functional activation mediated by K27 or other linkages. In contrast, RING ligases act as scaffolds, directly transferring ubiquitin from the E2 enzyme to the substrate. The discovery of the RING ligase SYVN1 promoting K27-linked ubiquitination of GSDMD illustrates a crucial point: the type of E3 ligase does not necessarily dictate the type of ubiquitin chain but rather determines the mechanism of transfer. Despite the significant strides in our understanding of Gasdermin ubiquitination, numerous questions remain. Why do cells need E3 ligases with opposing effects on pyroptosis? What are the specific signals controlling the expression and activity of these E3 ligases in different cell types and disease states? How do different ubiquitin chain types, beyond K27 and K48, influence Gasdermin biology? Further research, utilizing advanced proteomics, genetic screening, and structural biology, will be essential to unraveling these complexities.

From promoting pyroptotic activity through non-degradative linkages to triggering degradation of GSDMD/GSDME to prevent unchecked inflammation, the ubiquitin system adds layers of precision and control. By targeting the E3 ligases that orchestrate GSDMD/GSDME ubiquitination, we can fine-tune the inflammatory response in a wide range of diseases, marking a new era of targeted therapies for inflammatory metabolic diseases, including cardiovascular disease and cancer.
